# Atrioventricular accessory pathway unmasked by heart valve replacement

**DOI:** 10.1111/anec.12911

**Published:** 2021-11-16

**Authors:** Yanli Long, Yi Li, Zhibing Lu

**Affiliations:** ^1^ Department of Cardiothoracic Surgery Wuhan Asia Heart Hospital Affiliated to Wuhan University of Science and Technology Wuhan China; ^2^ Division of Cardiology Department of Internal Medicine Institute of Myocardial Injury and Repair Zhongnan Hospital of Wuhan University Wuhan University Wuhan China

**Keywords:** accessory pathway, intermittent preexcitation, valve replacement, ventricular tachycardia

## Abstract

A 50‐year‐old male patient with a history of severe valvular regurgitation underwent mitral and aortic valve replacement surgery 3 months ago. Preoperative 12‐lead electrocardiogram presented atrial flutter (AFL) and atrial fibrillation. AFL complicated with ventricular pre‐excitation was observed on current admission. The potential mechanisms underlying these changes were considered multifaceted, and valve replacement procedure may be a rare incentive factor.

## 1 CASE PRESENTATION

**FIGURE 1:**
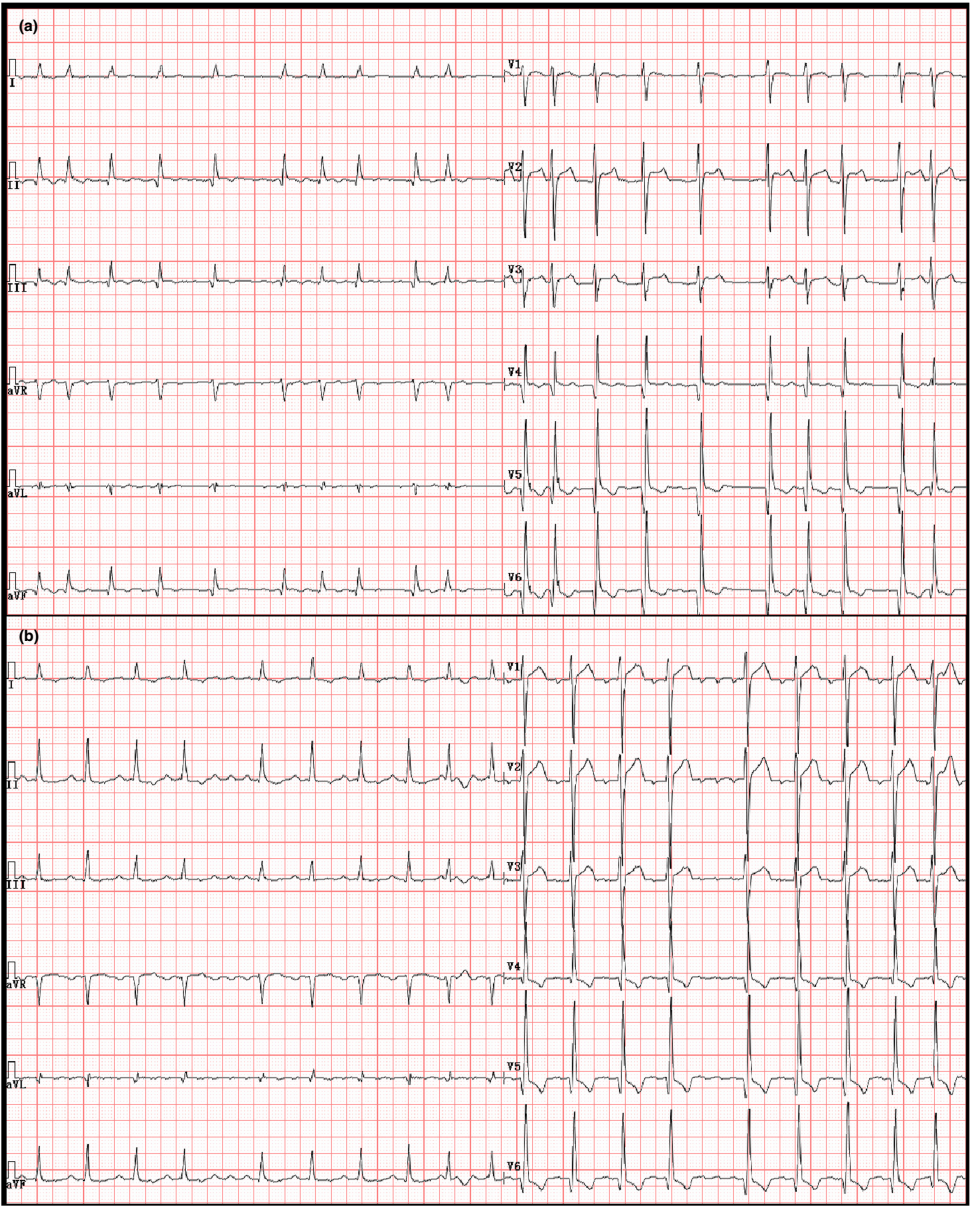
(a) Electrocardiogram (ECG) taken before valve replacement surgery revealed atrial fibrillation with *Q* waves in leads V_4_–V_6_. (b) ECG taken on 1 day postoperative indicated atrial flutter (3:1–5:1)

A 50‐year‐old male patient visited the ER for a week of palpitation, chest discomfort, and shortness of breath. He underwent mitral valve and aortic valve replacement surgery for severe valvular regurgitation 3 months ago. Preoperative 12‐lead electrocardiogram (ECG)‐presented atrial fibrillation (AF) with *Q* wave in leads V_4_–V_6_ (Figure 1a) and 24‐h ECG (Holter monitor) revealed alternation of AF and AFL. The patient underwent mitral and aortic valve replacements under cardiopulmonary bypass. The prosthetic valves used in the operation were the Edward biological valve and the Edward mechanical valve, respectively. ECG taken on Day 1 postoperatively showed AFL (3:1–5:1; Figure 1b). The patient received β receptor blocker therapy to control heart rate after the last discharge.

**FIGURE 2:**
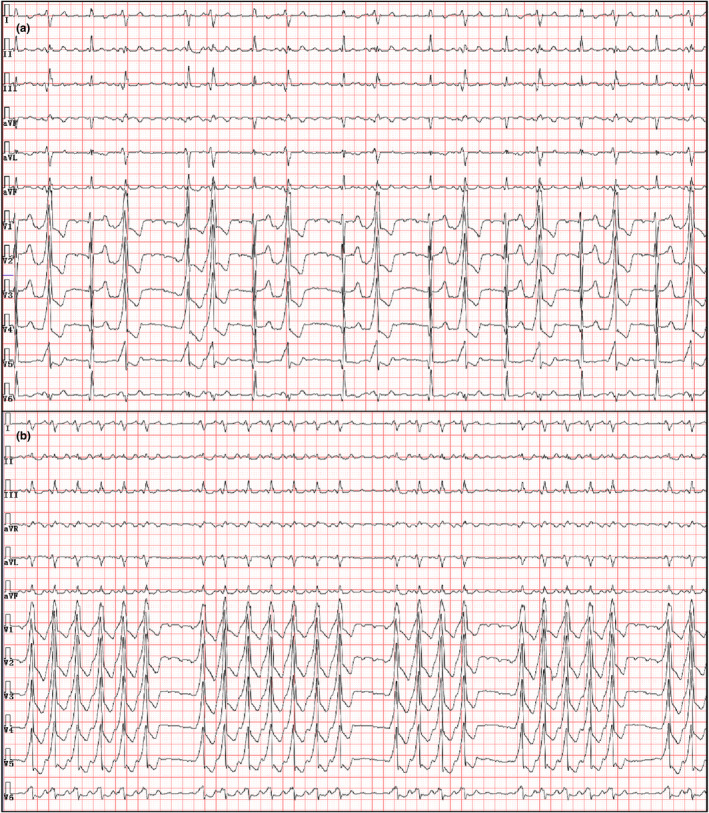
(a) Emergency electrocardiogram (ECG)‐presented intermittent wide complex beats as bigeminy. (b) ECG recorded after using metoprolol and deslanoside. Irregular wide complex tachycardia was noted

**FIGURE 3:**
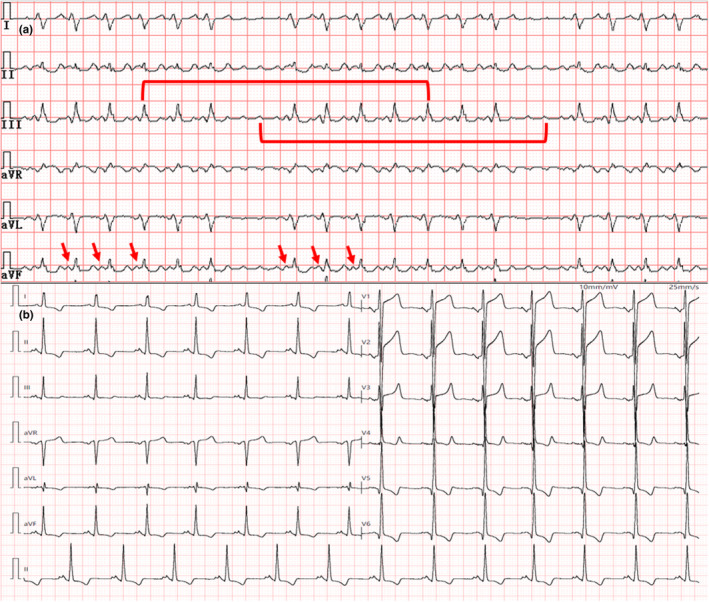
(a) When the atrial flutter anterograde through the accessory pathway, the interval between the peaks of any two QRS complexes (upper red line segment) always corresponded to the peaks of the two flutter waves (lower red line segment), and the morphology of flutter before each wide QRS complex was consistent. (b) Electrocardiogram recorded after atrial flutter and accessory pathway ablation

Current physical examination: Heart rate was 142 bpm with irregular rhythm; blood pressure was 95/68 mmHg; and the sound of mechanical valve can be detected in the aortic and mitral valve auscultation area. The characteristics of emergency ECG‐presented atrial rhythm (Figure 2a) were atypical AFL and bigeminy of premature ventricular contraction (PVC). Considering palpitation and symptomatic PVC, metoprolol and deslanoside were used to control ventricular rate. However, the patient's clinical condition aggravated and the ECG transformed into an irregular wide complex tachycardia (Figure 2b). Some extra characteristics of wide QRS complex included the following: (1) Interval between flutter wave and QRS complex was constant and shorter in wide QRS complex. (2) Divider test shown in Figure 3a indicated atrioventricular association. We considered the diagnosis of ECG was AFL complicated with intermittent ventricular pre‐excitation, rather than ventricular tachycardia with exit block. The patient received further electrophysiological (EP) study, and after the AFL ablation, ventricular pre‐excitation was observed in sinus rhythm. The AP was finally eliminated at the free wall of mitral valve with no AFL and wide QRS appeared in the Holter test before discharge (Figure 3b).

## 2 DISCUSSION

The main finding of this case is that cardiac surgery may be a rare inducing factor to unmask the potential AP in normal heart conduction. Previous reports mainly focused on the new onset of ventricular pre‐excitation following cardiac surgeries of correcting congenital anatomic abnormalities, such as Ebstein's anomaly, tricuspid atresia, and ventricular septal defect (VSD), which commonly complicated with AP (Ai et al., 1998; Chang et al., 2015; Hager et al., 2005; Peinado et al., 2007), while ventricular pre‐excitation following valve replacement is rarely described. Simmers TA and Gopinathannair R respectively reported cases of right AP unveiled following tricuspid valve replacement (Gopinathannair et al., 2013; Simmers et al., 2006). In our case, the AP was ablated on the mitral valve, which is consistent with the position of the valve procedure. Hence, we suspected that the exposure of the AP may be associated with valve replacement surgery.

Some potential reasons for the mechanism of pre‐excitation following valve replacement procedure could be summarized as follows: First, since most APs were anatomically adjacent to the atrioventricular annulus, surgical procedure, such as innate valve incision and prosthetic valve suture, may change the structure of AP or surrounding tissues. As a result, the anterograde conductivity of AP could be increased by the source–sink relationship improved (Dhein et al., 2014). Moreover, the growth of myocardial cells or the new presence of electrical conduction through the suture line, or so called “acquired bypass tract” (Peinado et al., 2007), is another notable reason, which was mainly observed following Fontan procedure and orthotopic heart transplantation. Furthermore, valve replacement operation, particularly in sutureless AVR procedure, may have potential lesion to atrioventricular conduction system and lead to atrioventricular block (Gonzalez et al., 2019; Simmers et al., 2006). The use of extracorporeal circulation and direct mechanical trauma can induce transient myocardial injury and may cause conduction abnormality (Clay‐Weinfeld & Callans, 2019). At last but not the least, perioperative drug applications should not be ignored. In previous studies, anesthetic drugs have been confirmed to potentially alter the conduction in the normal and AP (Staikou et al., 2018). β receptor blocker and digitalis, on the one hand, can control the ventricular rate in our case. On the other hand, it can unmask AP by decreasing the conduction of atrioventricular node (AVN). The explanation for the absence of ventricular pre‐excitation on the day after surgery may be associated with transient myocardial edema and injury, which may decrease the excitability and conductivity of AP. With the recovery of cardiac function and use of antiarrhythmic drugs, the typical ventricular pre‐excitation occurred.

The inspiration of this case is that making accurate differential diagnosis of wide QRS complex arrhythmia is particularly significant because the therapeutic principle is quite different, even opposite. If this case was misdiagnosed as ventricular bigeminy or ventricular tachycardia with an exit block while antiarrhythmic drugs were thus administrated, the degree of pre‐excitation and rapid ventricular rate may aggravate because of the further suppression of AVN. In our case, duration between the wide QRS complex and the flutter wave proceeded was equal in different ECG segments. Afterward, divider test (Figure 3a) indicated that the relation between the long RR interval and FL‐FL interval was integer multiple. None of the above supported atrioventricular dissociation (Surawicz et al., 2001), and thus, ventricular tachycardia with an exit block could be ruled out. However, lacking of the Holter result before valvular surgery could be a flaw during this clinical course.

## 3 CONCLUSION

We presented a case of accessory pathway unmasking following valve replacement surgery. The mechanisms mainly included changes in source–sink relationship and impairment of conduction system caused by surgical procedure and antiarrhythmic drug use. Accurate ECG investigation may contribute to make differential diagnosis and appropriate treatment strategy.

## CONFLICT OF INTEREST

The authors declared that they have no conflicts of interest to this work. We declare that we do not have any commercial or associative interest that represents a conflict of interest in connection with the work submitted.

## AUTHOR CONTRIBUTIONS

Conceptualized the Study, provided software and resources, performed formal analysis, and wrote the original draft: Y. Li. Designed methodology, and wrote and revised the manuscript: ZL. Investigated the study, visualized the data, and administered the project: Y. Long. Validated and curated data: Y. Li.

## 4 ETHICAL APPROVAL

The study complied with the edicts of the Declaration of Helsink (World Medical Association, 2013) and was approved by the patient and his family. Given that this is a retrospective case report, informed consent was waived.

## Data Availability

This is an open access article under the terms of the Creative Commons Attribution License, which permits use, distribution and reproduction in any medium, provided the original work is properly cited.
